# Impact of a Functional Dairy Powder and Its Primary Component on the Growth of Pathogenic and Probiotic Gut Bacteria and Human Coronavirus 229E

**DOI:** 10.3390/ijms25179353

**Published:** 2024-08-29

**Authors:** Vu Bao Dang, Muhammad A. Alsherbiny, Ruohui Lin, Yumei Gao, Chunguang Li, Deep Jyoti Bhuyan

**Affiliations:** 1 NICM Health Research Institute, Western Sydney University, Penrith, NSW 2751, Australia; v.dang@westernsydney.edu.au; 2 Pharmacognosy Department, Faculty of Pharmacy, Cairo University, Cairo 11562, Egypt; muhammad.alsherbiny@pharma.cu.edu.eg; 3 Australian Dairy Park Pty Ltd., 120 Frankston Gardens Drive, Carrum Downs, VIC 3201, Australia; ruohui.lin@adp-group.com.au; 4 Ausnutria Pty Ltd., 25-27 Keysborough Avenue, Keysborough, VIC 3173, Australia; linda.gao@adp-group.com.au; 5 School of Science, Western Sydney University, Penrith, NSW 2751, Australia

**Keywords:** functional milk product, gut bacteria, probiotic, proteomics, human coronavirus 229E, in vitro digestion

## Abstract

Milk boasts an array of potent bioactive compounds, such as lactoferrin (Lf), immunoglobulins, and functional proteins, all delivering substantial therapeutic benefits. In this study, Immune Powder (a functional dairy formulation) and its primary component called Fractionated Milk Protein (FMP) containing Lf, zinc, and immunoglobulins and formulated by Ausnutria Pty Ltd. were evaluated for their potential broad-spectrum pharmacological activity. In particular, this study investigated the antibacterial (against pathogenic *Escherichia coli*), prebiotic (promoting *Lactobacillus delbrueckii* growth), anti-inflammatory (inhibition of NO production in RAW264.7 macrophages), and antiviral (against human coronavirus 229E) effects of the samples. In addition, the impact of simulated gastric digestion on the efficacy of the samples was explored. LCMS-based proteomics was implemented to unveil cellular and molecular mechanisms underlying antiviral activity. The Immune Powder demonstrated antibacterial activity against *E. coli* (up to 99.74 ± 11.47% inhibition), coupled with prebiotic action (10.84 ± 2.2 viability fold-change), albeit these activities diminished post-digestion (*p* < 0.01). The Immune Powder effectively mitigated NO production in lipopolysaccharide-stimulated RAW264.7 macrophages, with declining efficacy post-digestion (*p* < 0.0001). The Immune Powder showed similar antiviral activity before and after digestion (*p* > 0.05) with up to 3-fold improvement. Likewise, FMP exhibited antibacterial potency pre-digestion at high concentrations (95.56 ± 1.23% inhibition at 125 mg/mL) and post-digestion at lower doses (61.82 ± 5.58% inhibition at 3906.25 µg/mL). FMP also showed enhanced prebiotic activity post-digestion (*p* < 0.0001), NO inhibition pre-digestion, and significant antiviral activity. The proteomics study suggested that the formulation and its primary component shared similar antiviral mechanisms by inhibiting scavenger receptor binding and extracellular matrix interaction.

## 1. Introduction

Milk, a nutritious lacteal secretion from healthy milch animals, is a vital component of the human diet globally. It contains various bioactive compounds, such as lactoferrin (Lf) [1], oligosaccharides, immunoglobulins, and functional proteins [2], contributing to its antimicrobial, antioxidant, and immunomodulatory functionalities beyond its primary role in nutrition [3]. The exploration of these functional components to develop novel dairy formulations is underway, with some already integrated into commercially available products [3].

Milk is a significant source of Lf [1], a multifunctional iron glycoprotein known for its anti-inflammatory and broad-spectrum antimicrobial activity against bacteria, fungi, and viruses [4,5,6]. The antimicrobial activity of Lf was attributed to its direct membrane-binding ability, damaging Gram-negative bacterial membranes and inhibiting bacterial colonisation or biofilm formation [7]. Lf has also been shown to inhibit the initial stages of infection by several viruses by binding directly to the virus particles or binding to docking or receptor sites for the virus on target mammalian cells [8,9,10]. Lf and zinc (Zn) have been recently reported for their potential role against SARS-CoV-2, primarily due to their antiviral, immunomodulatory, and anti-inflammatory effects [11,12]. Notably, a study revealed that bovine Lf binds to heparan sulphate proteoglycans of SARS-CoV-2, thereby blocking viral attachment to the host cell [13].

Zinc ions (Zn^2+^) are essential dietary trace metals for functions in the human body and have been found to exhibit promising antibacterial and antiviral properties. Zn^2+^ is believed to interfere with bacterial cell membrane integrity, DNA replication, and protein synthesis [14,15]. Zn^2+^ can also induce oxidative stress, leading to lipid peroxidation and membrane damage, resulting in β-lactamase enzyme inhibition, intracellular protein inactivation, DNA damage, and eventually cell death [15]. Additionally, Zn^2+^ was found to impede viral replication by inhibiting the activity of RNA polymerase and viral protease [16,17]. Zn ions were also shown to inhibit the replication of several viruses, including rhinoviruses, herpes simplex virus, and human immunodeficiency virus [16,17]. Additionally, Zn ions enhanced the immune system’s response to viral infections by increasing the production of interferons and natural killer cells [16,17].

Lactoperoxidase (LP) is an enzyme found in milk that participates in an unspecific humoral immune response through an inhibitory system called the LP system, which consists of SCN^−^ and H_2_O_2_ [18]. The LP system has strong inhibitory action against various pathogens, namely *Escherichia coli*, *Listeria monocytogenes*, and *Salmonella typhimurium* [19]. Its wide-spectrum antibacterial properties have been shown to control bacterial growth in diverse types of food, including dairy, fruit, vegetable, and meat [19].

Ausnutria Pty Ltd. (APL), a major Australian dairy powder manufacturer, has developed a novel functional dairy product containing Lf, Zn, and immunoglobulins called Immune Powder (Table S2 Supplementary File S1). This study investigated Immune Powder and its primary component manufactured by Saputo Australia, called Fractionated Milk Protein (FMP, a blend of bioactive proteins isolated from skim milk; Table S1, Supplementary File S1). Our investigation was centred on comprehending the antibacterial (targeting enterotoxigenic *E. coli*), prebiotic (enhancing *Lactobacillus delbrueckii* growth), and anti-inflammatory (in RAW264.7 macrophage cells) effects of the formulation and FMP along with their potential role in suppressing the cytopathic effects of human coronavirus 229E (HCoV-229E). The HCoV-229E is a less severe human coronavirus that causes mild-to-moderate upper-respiratory tract illnesses, including the common cold [20] and has recently gained attention as a model for performing preclinical screening, and designing antiviral agents, and understanding the host immune response to coronavirus infection [21]. Furthermore, we examined how the efficacy of the formulations could be impacted by in vitro gastric digestion. In addition, bottom-up quantification proteomics analysis was also performed to understand the cellular and molecular mechanisms of the potential antiviral activity of Immune Powder and its primary component, FMP.

## 2. Results and Discussion

### 2.1. Antibacterial Activity of the Immune Powder and FMP on the Growth of Pathogenic E. coli before and after In Vitro Digestion

**Figure 1:**
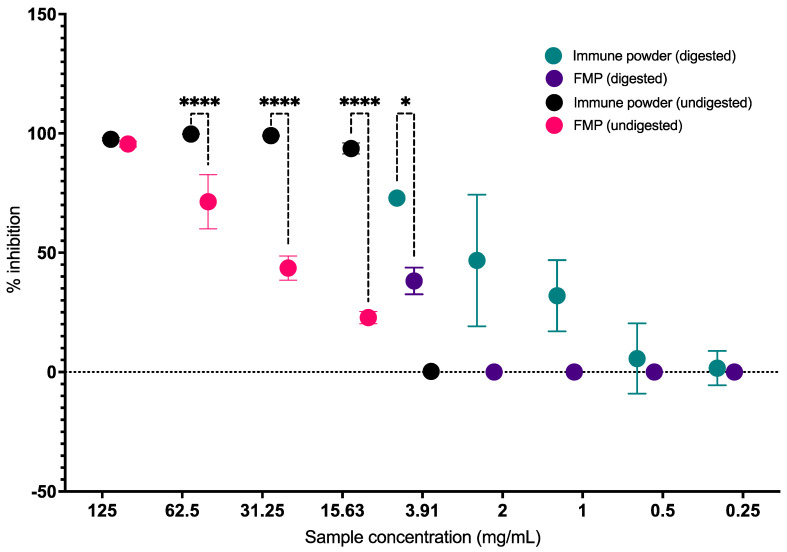
Antibacterial activity of the digested and undigested Immune Powder and Fractionated Milk Protein (FMP) samples against *E. coli.* Asterisks (*) signs indicate significant differences (*p* < 0.05) between the treatment groups according to two-way ANOVA (**** indicates *p* ≤ 0.0001).

The undigested Immune Powder sample, ranging in concentration from 15.63 to 125 mg/mL, and the FMP sample at a concentration of 125 mg/mL exhibited a growth inhibition rate of over 90% against the gastrointestinal pathogen *E. coli* (Figure 1). The study revealed that Immune Powder exhibited greater efficacy compared to FMP in suppressing the viability of *E. coli* within the concentration range of 15.63–62.5 mg/mL (*p* < 0.05). This enhanced effectiveness can be attributed to the inclusion of Lf and Zn in the composition of the Immune Powder. The antimicrobial properties of Lf are well-established, with studies showing its ability to inhibit the growth of a wide range of bacteria, viruses, and fungi [22], primarily due to its ability to sequester iron and interact between its positively charged surface with cations or anionic molecules, which are essential for the survival of many pathogens [22]. The antibacterial effect against pathogenic *E. coli* may also be attributed to LP in the formulation [19]. Similarly, the antibacterial activity of Zn is well-investigated against a wide range of Gram-positive and Gram-negative bacteria, including *E. coli* [23,24]. No antibacterial activities of the undigested Immune Powder and FMP were observed below 15.63 mg/mL.

The digested samples were also evaluated at a wide range of concentrations (0.0156–125 mg/mL) against *E. coli*. At high doses (in the 7.81–125 mg/mL range) of the Immune Powder and FMP, the assay parameters were impacted potentially due to the low pH caused by electrolyte contents from in vitro digestion. Low pH strongly reduces resorufin fluorescence [25], giving false readouts for cell viability. In the Alamar blue assay, the reduction of resazurin salt to resorufin via cellular dehydrogenase activity indicates cell viability [26]. Therefore, the antibacterial activity of the Immune Powder and FMP in the 7.81–125 mg/mL range could not be quantified accurately. The digested Immune Powder showed a 72.94% inhibition rate against *E. coli* at a concentration of 3.91 mg/mL (Figure 1). FMP also exhibited antibacterial properties against *E. coli* at a concentration of 3.91 mg/mL, resulting in a reduction in bacterial growth by 38.18%*,* and no antibacterial activity was observed at lower concentrations. Overall, the digested Immune Powder showed a significantly higher level of antibacterial activity against *E. coli* than the undigested Immune Powder and digested FMP within the concentration range of 1.00–3.91 mg/mL (*p* < 0.05). The enhanced antibacterial efficacy of Immune Powder after in vitro digestion may be attributed to the generation of lactoferricin through the enzymatic breakdown of Lf using pepsin [27]. A previous study of enzymatic hydrolysates of bovine Lf exhibited a wide range of antibacterial effects, effectively preventing the proliferation of various Gram-negative and Gram-positive bacteria [28]. Notably, the study indicated that lactoferricin has proven effective against strains that display resistance to native Lf, which showed a minimum of 8-fold higher antibacterial efficacy than undigested Lf when tested against all strains [28].

### 2.2. Prebiotic Activity of the Immune Powder and FMP on the Growth of L. delbrueckii before and after In Vitro Digestion

**Figure 2:**
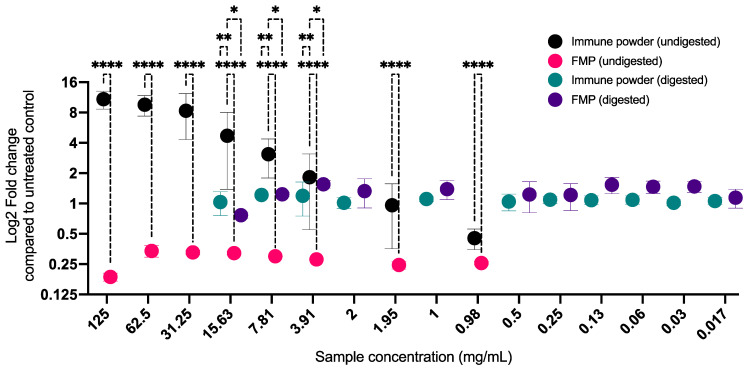
Effect of the Immune Powder and Fractionated Milk Protein (FMP) samples on the growth of *Lactobacillus delbrueckii* before and after in vitro digestion. Asterisks (*, **, and ****) signs indicate significant differences (*p* < 0.05, 0.01, and 0.0001, respectively) between the treatment groups according to two-way ANOVA with Tukey multiple comparison correction. The fold-change values are calculated based on the untreated control (increase or decrease compared to the untreated control). A fold-change value of over 1 (Log2FC of 0) indicates more cell growth than the untreated control.

A broad concentration range of 0.97–125 mg/mL was used to assess the prebiotic activity of the undigested Immune Powder and FMP. The undigested Immune Powder showed potent prebiotic activity by enhancing the growth of *L. delbrueckii* by over 10-fold compared to the untreated control (Figure 2), and particularly between 15.63 and 125 mg/mL, the undigested Immune Powder showed significantly higher prebiotic activity than the undigested FMP (*p* < 0.05). The greater prebiotic efficacy of undigested Immune Powder can be attributed to the constituents of full cream milk powder (Supplement Table S2), which contains lactose and protein, besides the standard composition of nutrient broth, which significantly facilitated the proliferation of *L. delbrueckii*. Another explanation for the observed inhibition of *L. delbrueckii* growth by the undigested FMP could be the absence of lactose in the sample. FMP primarily consists of a substantial quantity of LP (ranging from 25 to 70% *w*/*w*; Supplement Table S1). In conjunction with SCN− and H_2_O_2_, LP forms the lactoperoxidase system, which effectively inhibits the proliferation of Gram-positive, catalase-negative bacteria, such as streptococci and lactobacilli [29].

The prebiotic activity of the in vitro digested samples was also assessed across the same broad spectrum of concentrations, from 0.0156 to 125 mg/mL. When high doses of Immune Powder and FMP were applied (7.81 mg/mL–125 mg/mL), the low pH caused by the electrolyte contents from in vitro digestion influenced the assay parameters, as explained above. Hence, their prebiotic activity within that range could not be quantified accurately. Following in vitro digestion, FMP exhibited a growth enhancement of *L. delbrueckii* at a concentration of 3.92 mg/mL, resulting in a 1.55-fold increase compared to the untreated control. Similarly, the Immune Powder demonstrated a growth enhancement of 1.22-fold at a concentration of 7.81 mg/mL compared to the untreated control, as shown in Figure 2. However, after in vitro digestion, the Immune Powder in the 3.91–15.63 mg/mL range displayed significantly lower prebiotic activity than its undigested counterpart (*p* > 0.05). It should be noted that following in vitro gastric digestion, both the Immune Powder and FMP exhibited comparable prebiotic activity with no significant differences observed (*p* > 0.05). The significant difference in prebiotic activity of undigested and digested FMP (*p* < 0.0001) could be attributed to the production of amino acids and the inactivation of LP due to gastric digestion [30].

### 2.3. Anti-Inflammatory Activity of the Immune Powder and FMP before and after In Vitro Digestion

**Figure 3:**
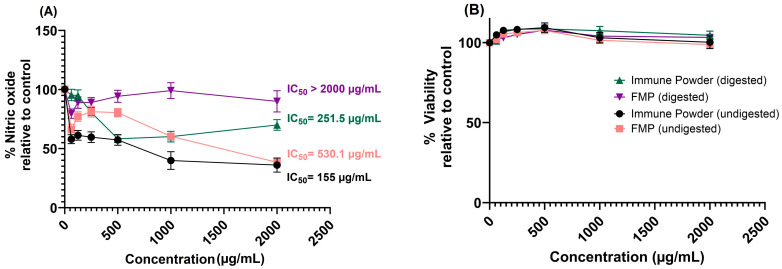
Effect of the Immune Powder and Fractionated Milk Protein (FMP) samples before and after in vitro digestion on nitric oxide production (**A**) and cell viability (**B**) in the lipopolysaccharide-stimulated murine RAW264.7 macrophages compared to control. Half-maximal inhibitory concentration (IC_50_) displays the sample concentration at which half of the nitric oxide production is inhibited. Data expressed as mean ± standard error mean (n = 3).

The anti-inflammatory activity of the Immune Powder and FMP samples, both undigested and digested, was assessed in murine RAW264.7 macrophage cells stimulated with lipopolysaccharides (LPS). The digested and undigested samples of Immune Powder and FMP (62.5–2000 µg/mL) showed no significant cell toxicity, as indicated by a mean cell viability of over 95% (Figure 3). Both digested and undigested Immune Powder showed dose-dependent anti-inflammatory activity by inhibiting nitric oxide (NO) production (Figure 3). Undigested FMP also inhibited the production of NO at concentrations above 1000 µg/mL; however, its activity was lost after in vitro gastric digestion (*p* < 0.0001). The undigested Immune Powder exhibited the greatest anti-inflammatory activity among the tested samples with an IC_50_ value of 155 µg/mL, possibly due to the presence of Lf and Zn in the formulation. Lf, one of the main components of the Immune Powder, has previously been shown to have anti-inflammatory properties potentially due to its interaction with negatively charged moieties (as the surface of Lf is positively charged), such as proteoglycans, on the surface of immune cells [31]. Similarly, reports have also demonstrated the anti-inflammatory activity of Zn [32,33]. Zn was found to decrease NF-κB activation and its target genes, such as TNF-α and IL-1β, and increase the gene expression of A20 and PPAR-α, the two zinc finger proteins with anti-inflammatory properties [33]. Based on the IC_50_ values, the anti-inflammatory activity of the samples can be categorised in the following order: undigested Immune Powder > digested Immune Powder > undigested FMP (Figure 3).

### 2.4. Immune Powder and FMP Cytotoxicity on MRC5 Lung Fibroblast Cells

**Figure 4:**
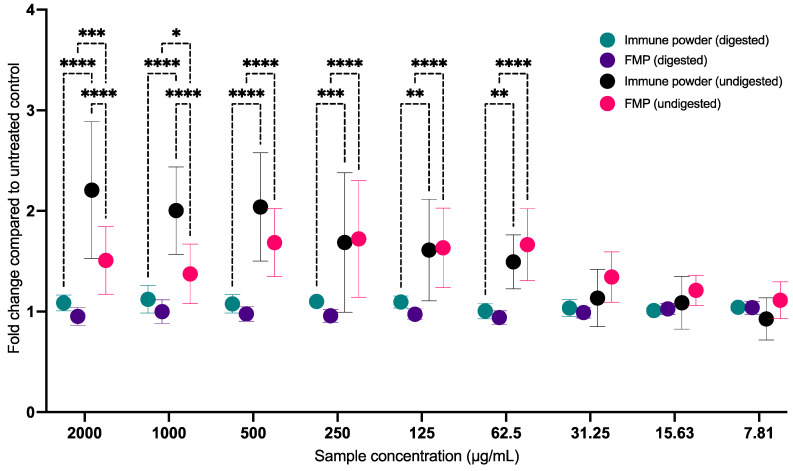
Effect of the Immune Powder and Fractionated Milk Protein (FMP) samples on the growth of the MRC5 lung fibroblast cells before and after in vitro digestion. The 1-fold change indicates similar cell growth compared to the untreated healthy control, whereas a fold-change value of more than 1 indicates cell growth enhancement compared to the untreated healthy control. Asterisks (*, **, ***, and ****) signs indicate significant differences (*p* < 0.05, 0.01, 0.001, and 0.0001, respectively) between the treatment groups according to two-way ANOVA with Tukey multiple comparison correction.

To assess the cytotoxicity towards the host MRC5 lung fibroblast cells, the Alamar blue assay was employed to evaluate both digested and undigested Immune Powder and FMP samples. The Immune Powder and FMP exhibited no cytotoxic effects within the 7.81–2000 µg/mL concentration range when tested against the MRC5 healthy (uninfected) lung cells. This observation was consistent before and after subjecting the cells to in vitro digestion, with cell viability exceeding 90% (Figure 4). Undigested Immune Powder, at concentrations of 2000, 1000, and 500 µg/mL, exhibited a significant increase in the growth of healthy lung cells. This increase was more than 2-fold and was statistically significant (*p* < 0.05) compared to the untreated control. This enhanced growth might be attributed to the presence of nutrients and growth factors in the formulation, compared to the digested Immune Powder and both digested and undigested FMP. In general, it was observed that the undigested Immune Powder demonstrated a significantly higher level of MRC5 cell growth-enhancing activity within the concentration range of 62.5–2000 µg/mL in comparison to the digested Immune Powder (*p* < 0.05).

### 2.5. Antiviral Activity of the Immune Powder and FMP against HCoV-229E before and after In Vitro Digestion

**Figure 5:**
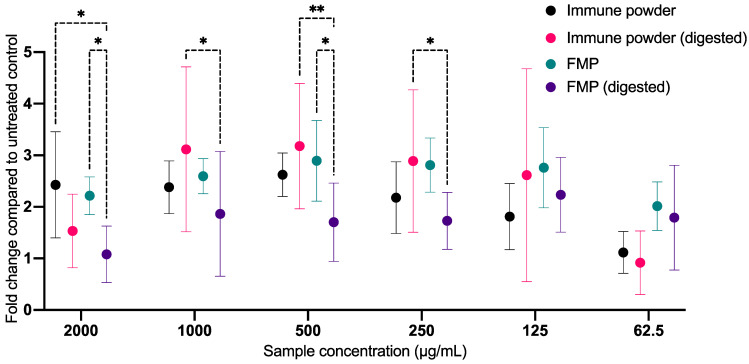
Antiviral activity of Immune Powder and Fractionated Milk Protein (FMP) against Human Coronavirus 229E before and after in vitro digestion. Asterisks (*) signs indicate significant differences (*p* < 0.05) between the treatment groups according to two-way ANOVA (** indicates *p* ≤ 0.01). The 1-fold change indicates similar cell growth compared to the untreated disease control, whereas a fold-change value of more than 1 indicates more healthy cells compared to the untreated disease control.

The host MRC5 lung fibroblast cells infected with HCoV-229E were used in this assay to determine the antiviral activity of the Immune Powder and FMP samples at different concentrations pre and post in vitro digestion. The Immune Powder and the FMP samples showed potential antiviral activity by protecting the lung cells from HCoV-229E infection before and after in vitro digestion (up to 3-fold healthier than the disease control; Figure 5). The in vitro gastric digestion process did not affect the antiviral activity of the Immune Powder as it showed similar activity before and after digestion (*p* > 0.05). The antiviral activity of Immune Powder could be ascribed to the potential synergy of its FMP component with other ingredients, such as Lf and Zn, in the formulation. Lf was previously shown to exhibit antiviral properties [22]. Likewise, Zn is known to exhibit various direct and indirect antiviral properties [34,35,36]. On the contrary, the antiviral activity of FMP was reduced by in vitro gastric digestion, especially at 2000 and 500 µg/mL (*p* < 0.05). The digested Immune Powder in the 250–1000 µg/mL range displayed significantly greater antiviral activity against the HCoV-229E infection than the digested FMP sample (*p* < 0.05). In the lower concentration range of 62.5–125 µg/mL, the digested and undigested Immune Powder and FMP showed similar antiviral activity (*p* > 0.05).

### 2.6. Proteomics Analyses of the Antiviral Activity

**Figure 6:**
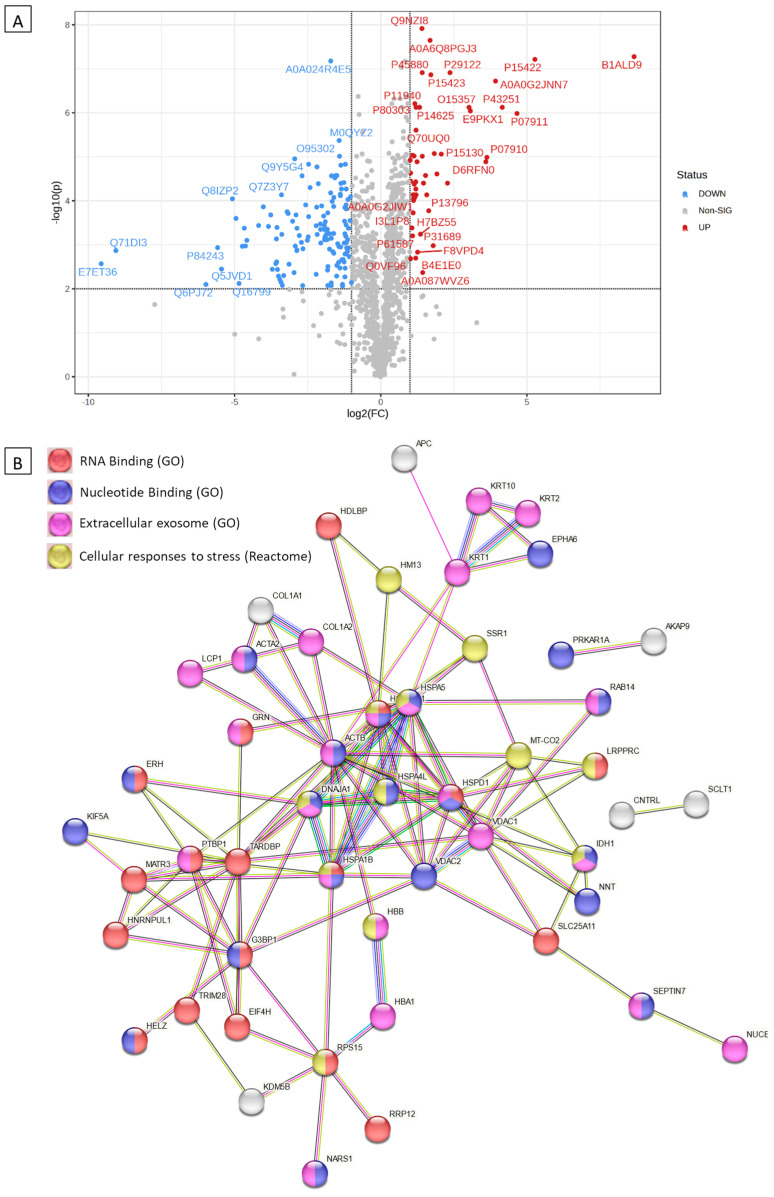
Differentially expressed proteins in remdesivir-treated HCoV-229E-infected MRC-5 cells compared to control (HCoV-229E-infected MRC-5 cells) and the corresponding over-represented pathways. (**A**) Volcano plot with an absolute log2 fold change ≥ 1 and *p*-value ≤ 0.05 cutoff for the identified proteins in remdesivir-treated HCoV-229E-infected MRC-5 lung fibroblast cells. (**B**) STRING network of the 62 differentially expressed proteins (fold change ≥ 2; *p* and Q values ≤ 0.01) in the remdesivir-treated HCoV-229E-infected MRC-5 cells compared to the controls. The minimum required interaction score was 0.40 (medium confidence), and red, green, blue, purple, light-blue, and black interaction lines indicate the presence of fusion, neighbourhood, co-occurrence, experimental, database, and co-expression evidence, respectively. The disconnected nodes were hidden in the network.

**Figure 7:**
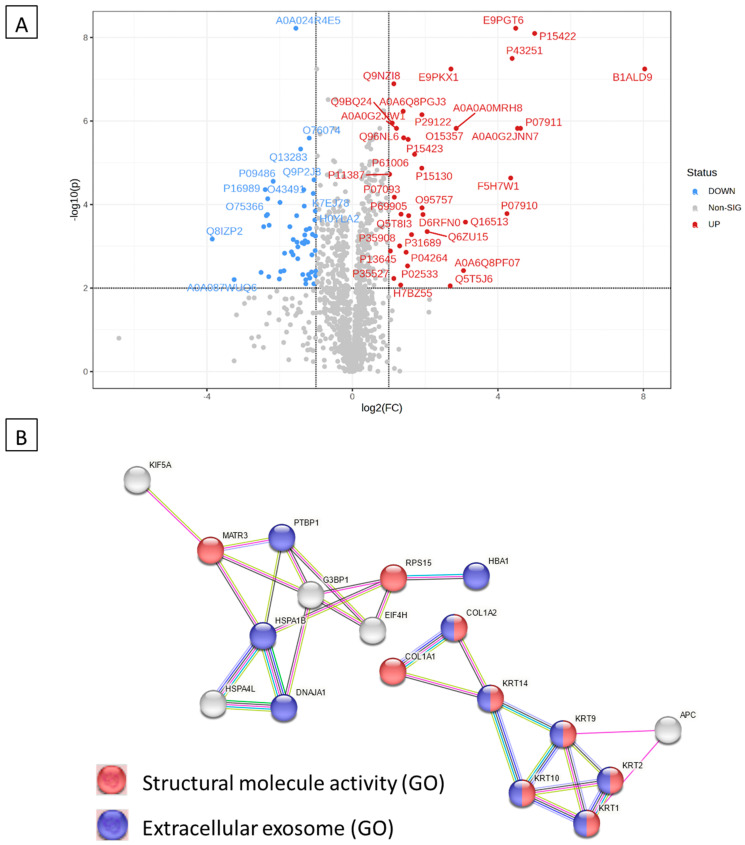
Differentially expressed proteins in Immune Powder-treated HCoV-229E-infected MRC-5 cells compared to control and the corresponding over-represented pathways. (**A**) Volcano plot with an absolute log2 fold change ≥ 1 and *p*-value ≤ 0.05 cutoff for the identified proteins in Immune Powder-treated HCoV-229E-infected MRC-5 cells. (**B**) STRING network of the 28 differentially expressed proteins (fold change ≥ 2; *p* and Q values ≤ 0.01) in the Immune Powder-treated HCoV-229E-infected MRC-5 cells compared to the controls. The minimum required interaction score was 0.40 (medium confidence), and red, green, blue, purple, light-blue, and black interaction lines indicate the presence of fusion, neighbourhood, co-occurrence, experimental, database, and co-expression evidence, respectively. The disconnected nodes were hidden in the network.

**Figure 8:**
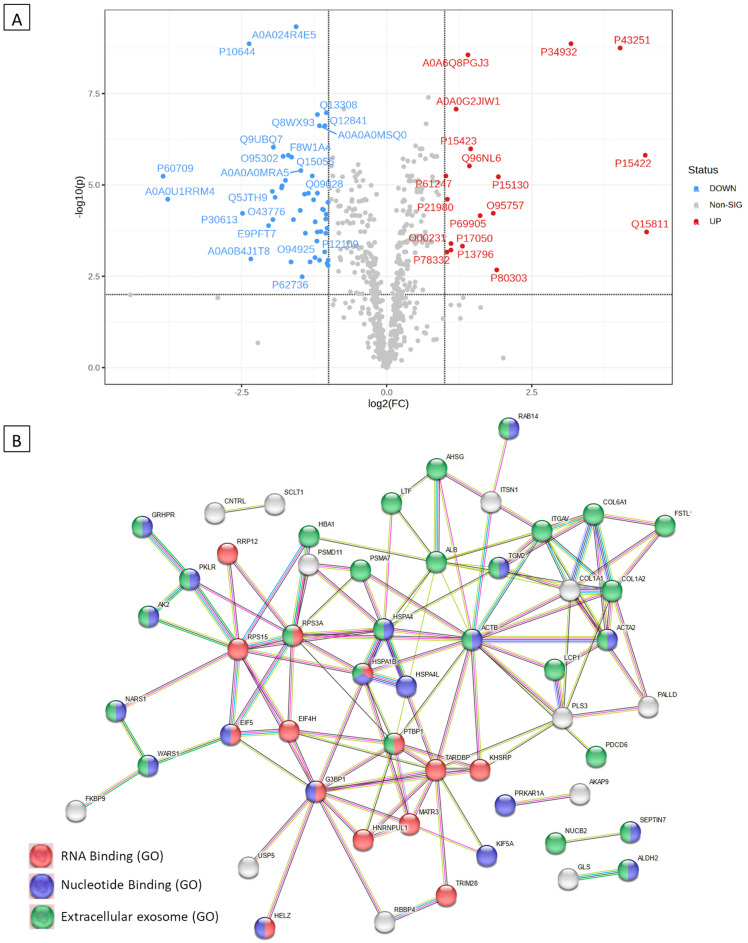
Differentially expressed proteins in Fractionated Milk Protein (FMP)-treated HCoV-229E-infected MRC-5 cells compared to control and the corresponding over-represented pathways. (**A**) Volcano plot with an absolute log2 fold change ≥ 1 and *p*-value ≤ 0.05 cut off for the identified proteins in FMP-treated HCoV-229E-infected MRC-5 cells. (**B**) STRING network of the 72 differentially expressed proteins (fold change ≥ 2; *p* and Q values ≤ 0.01) in the FMP-treated HCoV-229E-infected MRC-5 cells compared to the controls. The minimum required interaction score was 0.40 (medium confidence). Red, green, blue, purple, light-blue, and black interaction lines indicate the presence of fusion, neighbourhood, co-occurrence, experimental, database, and co-expression evidence, respectively. The disconnected nodes were hidden in the network.

The proteomics assay was used to identify patterns of co-regulated or unique pathways and link dysregulated protein sets to specific biological functions. Downregulated and upregulated proteins in samples treated with the standard antiviral drug remdesivir, Immune Powder, and FMP compared to HCoV-229E-infected samples (untreated disease control) are shown in Table 1. By annotating the gene IDs to biological pathways, enriched pathways were identified using the STRING network, as shown in Figure 6B, Figure 7B, and Figure 8B. The three samples (remdesivir, Immune Powder, and FMP) shared a few similar patterns of protein dysregulation, such as upregulated proteins in response to ER stress (*HSPA4L*, *HSPA1B*, and *DNAJA1*) and downregulated intra- and extra-cell signalling [37]. The elevated expression of ER stress-related proteins and heat shock proteins observed in remdesivir, Immune Powder, and FMP is consistent with previous findings, where the elevated proteins corresponded to SAR-CoV-2 virus infection [38].

Keratin gene family *KRT1*, *KRT2*, *KRT9*, *KRT10*, and *KRT14* were upregulated in samples treated with Immune Powder. The upregulation of the keratin gene family was observed previously in NHBE, A549, and Calu-3 cell lines infected with SARS-CoV-2 [39]. The proteins coded by these genes are related to the formation of hemidesmosome—a type of anchoring junction [39]. Although the hemidesmosome structure is essential for the passage of viruses from one cell to the basal lamina in the respiratory tract, the upregulation of the keratin gene family in this study may not help the virus replicate and may instead indicate viral infection.

All cells treated with remdesivir, Immune Powder, and FMP exhibited potential downregulation in *COL1A1* and *COL1A2* genes. The upregulation of *COL1A2* was previously observed under the expression of SARS-CoV N protein in 2BS cells [22,36]. Reactome pathway analysis of the downregulated genes showed that they may be attributed to the reduced scavenge by class A receptors pathway, GP1b-IX-V, collagen formation-related pathways, and the binding and uptake of ligands by scavenger receptors. Reduction in scavenger receptor-related pathways, in particular, the high-density lipoprotein (HDL) scavenger receptor B type 1 (SR-B1), which facilitates ACE2-dependent entry of coronavirus, suggested a mechanism of reducing virus and host cell receptor interaction [40]. 

In addition to the downregulation of *COL1A1* and *COL1A2* genes, the FMP-treated cells also experienced a significant reduction in *COL6A1* and *ITGAV* gene expression. These proteins are related to extracellular matrix (ECM) interactions, such as syndecan, ECM proteoglycans, ECM receptors, and integrins cell surface interaction pathways. The downregulation could prevent virus–host cell interaction and virus internalisation [41]. Previous research showed that during virus internalisation, syndecans colocalise with angiotensin-converting enzyme 2 (ACE2), suggesting a jointly shared internalisation pathway, and the virus internalisation was inhibited in the presence of syndecan inhibitors [42]. The downregulated syndecan interaction pathway suggested the deduction in virus internalisation ability. Inhibition of integrin cell surface interaction also contributes to blocking coronavirus cell entry, hence inhibiting productive infection of cells by SARS-CoV-2 [43].

The proteomics data suggested a shared antiviral mechanism of Immune Powder and FMP by preventing virus–host cell interaction. Lf and Zn were previously reported in the literature to have direct antiviral activity via blocking of viral receptors [22,36]. Lf has been shown to bind heparan sulphate co-receptors (HSPGs), which the SARS-CoV-2 spike attaches to, to enrich the local concentration before subsequently binding with the ACE2 receptor [13]. Therefore, by binding HSPGs, Lf indirectly prevents the virus attachment to the ACE2 receptor [13]. Zn-saturated Lf demonstrated a significant inhibitory effect on poliovirus type 1 infection, and the degree of inhibition was associated with the level of Zn saturation [44]. In addition, the Zn^2+^ cation is capable of dose-dependently suppressing ACE2 enzymatic activity, suggesting that Zn^2+^ could inhibit the interaction between virus protein S and the ACE2 receptor [36]. Although Zn^2+^ is also capable of inhibiting SARS-CoV-2 RNA polymerase and RNA-dependent RNA polymerase activity [36], the proteomic study did not show evidence of the dysregulation of these polymerases, suggesting there was no cellular Zn influx. Overall, the observed mechanisms of action of the Immune Powder and FMP could be attributed to the Lf and Zn activity on the ECM matrix but not intracellular activity. It also explains the differences in the antiviral activity of Immune Powder and FMP, where Immune Powder has an additional Zn component, resulting in higher antiviral efficacy compared to FMP, which only contains Lf.

## 3. Materials and Methodology

### 3.1. Cell, Viral, and Bacterial Culture

Gut pathogen *Escherichia coli* (ATCC 35401^TM^) was cultured and maintained in nutrient broth (Sigma-Aldrich, Bayswater, VIC, Australia). *Lactobacillus delbrueckii* (ATCC 9649^TM^) was cultured and maintained in Difco^TM^ Lactobacilli MRS Broth (BD, Macquarie Park, NSW, Australia). Both bacterial strains were incubated for 24 h prior to the assays at 37 °C (BD 23; Binder GmbH, Tuttlingen, Germany).

Medical Research Council cell strain 5 (MRC-5) is a diploid cell culture line composed of lung fibroblasts that are susceptible to viral infections [45], and is widely used for the production of viral vaccines. HCoV-229E (ATCC VR-740^TM^) and its host MRC-5 (ATCC CCL-171) lung fibroblast cells were purchased from the American Type Culture Collection (ATCC, Manassas, VA, USA) and cultured and maintained in Eagle’s minimum essential medium (EMEM; Lonza Australia Pty Ltd., Norwest, NSW, Australia) supplemented with 10% fetal bovine serum (FBS; Interpath, Somerton, VIC, Australia), nonessential amino acids, 1 mM of sodium pyruvate, 2 mM of L-glutamine (Lonza Australia Pty Ltd., Norwest, NSW, Australia), and 1% penicillin and streptomycin (Sigma-Aldrich, Bayswater, VIC, Australia) at 37 °C under a humidified atmosphere of 5% CO_2_ (In VitroCell ES; In vitro Technologies, Lane Cove West, NSW, Australia) as per the ATCC protocol.

The murine RAW264.7 (ATCC TIB-71^TM^) macrophage cells were cultured in Dulbecco’s modified Eagle’s medium (DMEM; Lonza Australia Pty Ltd., Norwest, NSW, Australia) supplemented with 5% FBS (FBS; Interpath, Somerton, VIC, Australia), and 1% penicillin–streptomycin (#P4333; Sigma Aldrich, Bayswater, VIC, Australia) at 37 °C in a humidified incubator containing 5% CO_2_ (In VitroCell ES; In vitro Technologies, Lane Cove West, NSW, Australia).

### 3.2. In Vitro Gastric Digestion of the Immune Powder and FMP

The in vitro gastric digestion of the Immune Powder and FMP was performed using a method described earlier by Minekus and Alminger [46]. The electrolyte stock solution was prepared using the information provided in Table S3 of Supplementary File S1. A mixture of porcine pepsin (3200–4500 U/mg protein) was used to prepare the porcine pepsin stock solution. The stimulated gastric digestion was performed by mixing five parts of liquid Immune Powder or FMP (250 mg/mL) with four parts of electrolyte stock solution, porcine pepsin stock solution, 0.3 M CaCl_2_, and water to prepare a final enzyme concentration of 2000 U/mL and 0.075 mM CaCl_2_. Prior to introducing the digestion mixture to the thermal shaker (MaxQ^TM^ 4000; Thermo Fisher Scientific, North Ryde, NSW, Australia) at 37 °C for 2 h, the pH of the mixture was adjusted to 3.0 using 1M HCl. Subsequently, the digestion process was stopped by freezing the mixture in a −20 °C freezer. The digested samples were then freeze-dried to obtain powdered extracts.

### 3.3. Determining the Antibacterial Activity of the Immune Powder and FMP on the Growth of Pathogenic E. coli before and after In Vitro Digestion

The antibacterial activity of the formulations was assessed against the common gut pathogen *E. coli,* which causes several human illnesses, including gastrointestinal and urinary tract infections, pneumonia, and meningitis [47]. Antimicrobial activity was measured using a resazurin dye-based assay to determine the oxidation level during cellular respiration, which is directly proportional to the number of viable cells [26]. Prior to the assay, the *E. coli* stock was adjusted to 0.5 McFarland standard (OD_530nm_ = 0.12–0.15). Using ultrasound (HWASHIN POWERSONIC420, Thermoline Scientific, Wetherill Park, NSW, Australia), the undigested and digested Immune Powder and FMP samples were dissolved in sterile distilled water for 10 min. The assay was conducted in 96-well plates; each well contained 10 µL of *E. coli* inoculum, 50 µL of the sample at various concentrations, 10 µL of resazurin salt (6.75 mg/mL), and nutrient broth until a final volume of 100 µL. The antibiotic ciprofloxacin (5 μg/mL) served as the positive control in the assay. The plates were incubated at 37 °C for 24 h. The fluorescence was then measured in a micro-plate spectrophotometer (BMG CLARIOstar, Melbourne, VIC, Australia) with an excitation wavelength of 530 nm, and an emission at 590 nm was recorded to quantify the viability. The viability in the treatment groups was calculated relative to the untreated negative control. 

### 3.4. Determining the Prebiotic Activity of the Immune Powder and FMP on the Growth of Probiotic Lactobacillus delbrueckii before and after In Vitro Digestion

*L. delbrueckii* is a probiotic bacterium commonly found in the gut as well as in yoghurt and several other probiotic foods [48]. The impact of the digested and undigested formulations on the growth of *L. delbrueckii* was determined to understand their potential prebiotic activity. *L. delbrueckii* inoculum was prepared by centrifuging and resuspending the stock culture in nutrient broth to obtain 0.5 McFarland standard (OD_530nm_ = 0.12–0.15) prior to the assay. The digested and undigested Immune Powder and FMP samples were dissolved in sterile distilled water using ultrasound for 10 min (POWERSONIC 420, Thermoline Scientific, NSW, Australia). The assay was conducted in a 96-well plate, each well contained 10 µL of *L. delbrueckii* inoculum, 50 µL of the sample at various concentrations, 10 µL of resazurin salt (6.75 mg/mL), and nutrient broth until a final volume of 100 µL. The assay used the antibiotic ciprofloxacin (5 μg/mL) as the positive control. The plates were incubated under aerobic conditions at 37 °C for 24 h. The fluorescence was then measured at an excitation wavelength of 530 nm in the microplate spectrophotometer, and an emission at 590 nm was recorded to quantify the degree of oxidation occurring during cellular respiration, which directly correlated with the number of live cells present in the wells [26].

### 3.5. Evaluation of the Anti-Inflammatory Activity of the Immune Powder and FMP before and after In Vitro Digestion

The anti-inflammatory activity of the digested and undigested Immune Powder and FMP at different concentrations was evaluated using a NO assay on the murine RAW264.7 macrophage cells, as per our previously described protocols [26,49]. Briefly, the NO production stimulated by 50 ng/mL of LPS in the RAW264.7 macrophage cells was measured via the total nitrite content using the Griess reagents (a mixture of an equal amount of 1% sulphanilamide in 5% phosphoric acid and 0.1% N-1-(naphthyl)ethylenediamine dihydrochloride). The potential cytotoxicity of the samples in the RAW264.7 macrophage cells was also evaluated using the MTT assay relative to the control [26].

### 3.6. Impact of the Immune Powder and FMP on the Viability of the MRC5 Lung Fibroblast Cells

The potential cytotoxicity of the digested and undigested samples on the host MRC5 lung fibroblast cells was determined using an Alamar blue assay [26]. The MRC5 cells were incubated in a 96-well plate to reach a confluence of 80% before adding samples and complete EMEM to achieve a final concentration ranging from 7.81 µg/mL to 2000 µg/mL. Complete EMEM (supplemented with 5% FBS) was the negative control. The plate was then incubated at 37 °C and 5% CO_2_ for five days. At the end of the incubation period, media were removed, and cells were incubated with 100 µL of 100 µg/mL resazurin salt in EMEM for 1–2 h at 37 °C and 5% CO_2_ to quantify cell viability. The fluorescence was then measured in the micro-plate spectrophotometer (BMG CLARIOstar, Melbourne, VIC, Australia) with an excitation wavelength of 530 nm, and an emission at 590 nm was recorded to quantify the cell viability relative to the negative control.

### 3.7. Evaluation of the Antiviral Activity of the Immune Powder and FMP against HCoV-229E

The effect of the Immune Powder and FMP before and after in vitro gastric digestion at different concentrations on the late stages of HCoV-229E infection was measured on the host MRC5 lung fibroblast cells using a cytopathic effect (CPE) assay based on the previously described protocol by Hu and Ma [50]. MRC5 cells were incubated with complete EMEM in a 96-well plate for 24–48 h at 37 °C in the presence of 5% CO_2_. Once the culture reached 80% confluence, the MRC5 cells were incubated with 100-fold diluted HCoV-229E stock in EMEM without FBS in the cell culture incubator (humidified, 5% CO_2_, 37 °C) for 2 h to allow virus absorption. Samples (with a final concentration ranging from 62.5 to 2000 µg/mL) and the standard positive control drug remdesivir (75 µM) were then added to the wells. Remdesivir stock solution was prepared by dissolving the powder in dimethyl sulfoxide (DMSO) to 2 mg/mL concentration (3.32 mM), and the aliquots were stored at −20 °C. The neutral red working solution was prepared by dissolving the neutral red powder in EMEM to the concentration of 50 µg/mL, then centrifuging at 3100× *g* for 10 min. The neutral red de-staining solution was prepared by adding 500 mL of ethanol (96%), 490 mL of nano pure water, and 10 mL of glacial acetic acid. The de-staining solution was mixed well by stirring for 15 min and stored at room temperature (20–30 °C) for up to 2 months. At the end of incubation, each well was stained with 100 µL of neutral red working solution for 2–4 h. The culture media were removed and washed twice with PBS and fully dried before de-staining with the de-staining solution on the plate shaker at 400 rpm for 10 min. Results were collected by measuring the absorbance of neutral red extract at 540 nm in the micro-plate spectrophotometer (BMG CLARIOstar, Melbourne, VIC, Australia).

### 3.8. Cellular and Molecular Mechanism of the Antiviral Activity

#### 3.8.1. Protein Extraction

The HCoV-229E-infected MRC5 cells were prepared in T75 flasks as described in Section 3.7 before adding Immune Powder (500 µg/mL final concentration), FMP (500 µg/mL final concentration), and the standard positive control drug, remdesivir (75 µM). Complete EMEM supplemented with 5% FBS was added to the untreated control. The flasks were incubated for five days prior to protein extraction. At the end of the incubation, the cell culture media were collected, followed by adding 0.25% *w*/*v* trypsin solution to each cell flask for 3 min at 37 °C. To neutralise trypsin, an equal volume of EMEM containing 10% FBS was added before combining it with the previously collected media. The cells were then centrifugated at 500× *g* for 5 min at room temperature. Subsequently, the resulting pellets were washed twice with ice-cold PBS and subjected to another round of centrifugation at 500× *g* for 5 min. In 100 µL of lysis buffer containing 1 µL of universal nuclease, the cell pellets were then reconstituted, along with a fully mass spectrometry-compatible Halt™ Protease and Phosphatase Inhibitor Cocktail, EDTA-Free (Thermo Fisher Scientific, Waltham, MA, USA). The cells were resuspended by pipetting 10–15 times to reduce the viscosity of the sample and left on ice for a duration of 20 min. The lysate was centrifuged at 14,000 rpm for 20 min at 4 °C, after which supernatant was collected.

#### 3.8.2. Protein Quantification

The Pierce™ Rapid Gold BCA Protein Assay Kit (#A53226; Thermo Fisher Scientific, Waltham, MA, USA) was used to measure the protein concentration of the cell lysate. This was performed in triplicate and compared to a bovine serum albumin (BSA) standard, according to the manufacturer’s instructions. In summary, a volume of 1 µL from each replicate of the samples was diluted at a ratio of 1:20 in water, along with 20 µL from each standard. Subsequently, this mixture was transferred into individual wells of a 96-well plate containing 200 µL of the working reagent per well. The samples were then diluted until they reached a concentration range of 20–2000 µg/mL. The plate was then thoroughly mixed on a plate shaker for 30 sec and then incubated at room temperature for 5 min. The absorbance was measured within 20 min at 480 nm using a microplate spectrophotometer (BMG CLARIOstar, Melbourne, VIC, Australia). The baseline absorbance value was subtracted from all measurements of standards and samples. The concentration of the samples was then determined by comparing them to the established BSA standard calibration curve. The samples were stored at −80 °C until further analysis.

#### 3.8.3. Preparation and Clean-Up of Peptides

EasyPep™ Mini MS Sample Prep Kit (Thermo Fisher Scientific, Waltham, MA, USA) was employed to perform chemical and enzymatic sample processing. Following the manufacturer’s protocol, the protein samples (100 µg) were adjusted to 100 µL using a lysis buffer in a micro-centrifuge tube. The solutions for reduction and alkylation, each measuring 50 µL, were combined, mixed gently, and incubated at 95 °C using a heat block for 10 min. The samples were cooled at room temperature, followed by the addition of 50 µL of the reconstituted trypsin/lys-C protease mixture and incubated with shaking at 37 °C for 3 h. After incubation, 50 µL of digestion stop solution was added and mixed gently before running the samples through peptide clean-up columns to eliminate hydrophilic and hydrophobic impurities. Clean peptide samples were dried using a vacuum centrifuge, resuspended in 100 µL of 0.1% formic acid in water, and carefully transferred into maximum recovery sample vials (Waters Corp, Milford, MA, USA) for LC-MS analysis.

#### 3.8.4. Label-Free Bottom-Up Quantification Proteomics Analysis via Nano-Ultra-High-Performance Liquid Chromatography Coupled with Quadruple Time-of-Flight Mass Spectrometry (Nano-UPLC-qTOF-MS)

For the analysis of tryptic peptides, a nanoACQUITY UPLC system (Waters Corp., Milford, MA, USA) was used in conjunction with a Synapt G2-S high-definition mass spectrometer (HDMS) (Waters Corp., Manchester, UK). The mass spectrometer operated in positive electron spray ion mode (ESI+), following a method that had been described earlier [49,51]. To ensure precise mass accuracy, a lock spray solution of 100 fg/mL Glu-fibrinopeptide B in a mixture of 50% aqueous acetonitrile and 0.1% formic acid was used.

The peptides chromatographic separation was performed in the nanoEase M/Z BEH C18 (1.7 μm, 130 Å, 75 μm × 100 mm, Waters Corp., Milford, MA, USA) coupled with a nanoEase M/Z Symmetry C18 Trap Column (100 Å, 5 µm, 180 µm × 20 mm, Waters Corp., Milford, MA, USA) at 40 °C. The mobile phase A and B solutions were Milli-Q water and acetonitrile containing 0.1% formic acid (LCMS grade, Merck, Germany), respectively. A constant injection volume of 1 µL at a flow rate of 300 nL/min was used for the entire duration of the 50 min gradient, where the samples were introduced into the trapping column at a flow rate of 5 μL/min with a mobile phase of 99% phase A for 3 min. The mobile phase B was initially set at 1% and gradually increased to 85% over 50 min using a gradient consisting of 10% B at 2 min, 40% B at 40 min, and 85% B at 42 min. The samples were stored at 4 °C and were injected in duplicate. The temperature of the ion source block was adjusted to 80 °C, while the capillary voltage was consistently maintained at 3 kV. The ions were obtained within a mass-to-charge ratio (*m*/*z*) range of 50 to 2000 with 0.5 sec scanning duration. The sample cone voltage and source offset were set at 30 volts, the nanoflow gas pressure was maintained at 0.3 Bar, the purge gas flow rate was 20 L/h, and the cone gas flow rate was also set at 20 L/h. The data-independent acquisition (DIA) method used in this study for sample acquisition employed the MSE multiplex mode. Data acquisition was conducted using MassLynx (version 4.1) Mass Spectrometry Software (Waters Corporation, Milford, MA, USA).

### 3.9. Data Processing

The MassLynx (version 4.1) data obtained were imported and processed using Progenesis QI software (version 2.0, Waters Corp., Milford, MA, USA). The alignment reference for QC samples was automatically selected, and peptides were identified against the UniProt human proteome database (May 2022 version) using the ion-accounting method with a maximum protein mass of 250 kDa. Using relative quantification with the Hi-N method (n = 3), the ion-matching requirements were established as follows: one fragment per peptide or one peptide per protein, in addition to three fragments per protein. Search tolerance criteria included auto-peptide and fragment tolerance and less than 4% FDR. Peptides that had an absolute mass error greater than 20 ppm or have been single-charged peptides were excluded from further analysis. Comparisons were made between the identified proteins in the treated groups and the control group to explore their cytotoxic potential. For each experimental design, proteins that had a *p*- and *q*- (adjusted *p*) value of at least 0.05, as determined via analysis of variance (ANOVA), and a |log2fold change| of ≥2 were considered significant. These proteins were included for further pathway analyses. The differentially expressed proteins identified through quantitative processing of the LC-MS/MS analysis of the proteome tryptic digestion were analysed using STRING (https://string-db.org/, accessed on 12 February 2023) [52], g:Profiler (https://biit.cs.ut.ee/gprofiler/gost, accessed on 12 February 2023) [53], Reactome (https://reactome.org/, accessed on 12 February 2023) [54], and IMPaLA (http://impala.molgen.mpg.de/, accessed on 30 April 2024) [55] to determine the specific pathways involved in the antiviral mechanism of Immune Powder and FMP. For multiple-testing corrections in the g:Profiler platform, the g:SCS algorithm was applied with an adjusted *p* value threshold of 0.05. The unprocessed and processed data were subsequently submitted to the ProteomeXchange Consortium through the PRoteomics IDEntifications (PRIDE) repository [56] with the following dataset identifier: 10.6019/PXD052339, Project accession: PXD052339, and Token: ZMONUGmsYz4r to be accessed via the following unique link: https://www.ebi.ac.uk/pride/review-dataset/42590c88f30949519fb027b601bc1d9b accessed on 28 August 2024.

### 3.10. Statistical Analyses

The GraphPad Prism software (version 8.0) was used to perform the one- and two-way ANOVA and the Tukey post hoc test to compare the means between the control and the treatment groups. Results were expressed as means ± SD. All experiments were performed at least in triplicate. In all tests, adjusted *p* < 0.05 value was used as the criterion for statistical significance. In the shotgun proteomics study, MetaboAnalyst 5.0 was used along with Progenesis QI for Proteomics for the statistical analysis of the quantified proteins across conditions.

## 4. Conclusions and Future Directions

Overall, Immune Powder and its primary component, FMP, showed potential antibacterial, antiviral, and anti-inflammatory properties in this study. Immune Powder also demonstrated notable antibacterial activity against *E. coli* before and after digestion and displayed prebiotic activity pre-digestion, which was reduced after digestion. Immune Powder effectively inhibited NO production in stimulated RAW264.7 macrophage cells, and the efficacy was reduced post-digestion. The potential antiviral activity of Immune Powder by protecting the lung cells from HCoV-229E infection before and after in vitro digestion could be attributed to its Lf, Zn, and FMP components and the potential synergistic interactions among these components. FMP exhibited significant antibacterial activity pre-digestion, which was enhanced post-digestion. FMP also displayed prebiotic activity after digestion and NO inhibition pre-digestion, but it was diminished post-digestion. FMP showed no cytotoxicity toward lung cells and protected them against HCoV-229E infection pre-digestion, with reduced efficacy post-digestion. FMP shared similarities in the antiviral mechanism with Immune Powder by inhibiting scavenger receptor binding and ECM interaction.

Future studies (such as peptidomics and metabolomics) are warranted to analyse the chemical changes resulting from digestion and gut microbial metabolism of Immune Powder and FMP. This will also explain the differences in the bioactivity observed between the digested and undigested samples. Other pathogenic bacteria commonly found in the gut, including *Enterococcus faecalis* and *Klebsiella species*, as well as the common cold and flu viruses (such as the Influenza virus and Rhinoviruses), can also be included in future studies to understand the broad-spectrum activity of Immune Powder. This study informs future studies to establish the efficacy of dairy formulations against other medically important pathogens, including SARS-CoV-2 and Influenza virus.

## Figures and Tables

**Table 1:** Significantly dysregulated proteins (adjusted *p*-value ≤ 0.01; Progenesis QIP-calculated maximum fold change ≥ 2) analysed via bottom-up label-free quantification proteomics in the HCoV-229E-infected MRC-5 lung fibroblast cells treated with remdesivir, Immune Powder, and Fractionated Milk Protein (FMP) compared to the untreated disease control (HCoV-2-infected MRC-5 cells).

Uniprot ID	HGNC Gene ID	Protein Name	Log2 Fold Change
Remdesivir-treated HCoV-229E-infected MRC-5 cells compared to control (HCoV-2-infected MRC-5 cells)			
Q5JVD1	*CNTRL*	Centriolin	−5.78
J3QS41	*HELZ*	Probable helicase with zinc finger domain	−3.59
P68871	*HBB*	Hemoglobin subunit beta	−3.35
K7EKL3	*GRN*	Progranulin (Fragment)	−3.32
A0A0U1RRM4	*PTBP1*	Polypyrimidine tract-binding protein 1	−2.79
P60709	*ACTB*	Actin_ cytoplasmic 1	−2.48
E9PFT7	*APC*	Adenomatous polyp	−2.04
O15031	*PLXNB2*	Plexin-B2	−1.95
Q5JTH9	*RRP12*	RRP12-like protein	−1.83
P52306	*RAP1GDS1*	Rap1 GTPase-GDP dissociation stimulator 1	−1.82
A0A0B4J1T8	*EPHA6*	Receptor protein-tyr	−1.81
A0A6I8PUA5	*AKAP9*	A-kinase anchor protein 9 (Fragment)	−1.81
A0A024R4E5	*HDLBP*	High-density lipoprotein-binding protein (Vigilin)_ isoform CRA_a	−1.71
P49221	*TGM4*	Protein-glutamine gamma-glutamyltransferase 4	−1.69
Q15056	*EIF4H*	Eukaryotic translation initiation factor 4H	−1.61
P62736	*ACTA2*	Actin_ aortic smooth muscle	−1.61
O43776	*NARS1*	Asparagine--tRNA ligase_ cytoplasmic	−1.60
Q9UBG0	*MRC2*	C-type mann	−1.57
A0A0R4J2E8	*MATR3*	Matrin-3	−1.50
A0A087WTA8	*COL1A2*	Collagen alpha-2(I) chain	−1.50
P10644	*PRKAR1A*	cAMP-dependent protein kinase type I-alpha regulatory subunit	−1.47
O95302	*FKBP9*	Peptidyl-prolyl cis-trans isomerase FKBP9	−1.41
O76074	*PDE5A*	cGMP-specific 3′_5′-cyclic ph	−1.37
K7EJ78	*RPS15*	40S rib	−1.35
Q8IWE2	*FAM114A1*	Protein NOXP20	−1.33
P42704	*LRPPRC*	Leucine-rich PPR motif-containing protein_ mitochondrial	−1.26
Q9H6A9	*PCNX3*	Pecanex-like protein 3	−1.23
Q13148	*TARDBP*	TAR DNA-binding protein 43	−1.23
P84090	*ERH*	Enhancer of rudimentary homolog	−1.22
Q13283	*G3BP1*	Ras GTPase-activating protein-binding protein 1	−1.21
Q9UBQ7	*GRHPR*	Glyoxylate reductase/hydroxypyruvate reductase	−1.20
Q13263	*TRIM28*	Transcription intermediary factor 1-beta	−1.18
A0A0A0MRA5	*HNRNPUL1*	Heterogeneous nuclear ribonucleoprotein U-like protein 1	−1.18
P02452	*COL1A1*	Collagen alpha-1(I) chain	−1.18
P61106	*RAB14*	Ras-related protein Rab-14	−1.17
E7EPK1	*SEPTIN7*	Septin-7	−1.07
O75874	*IDH1*	Isocitrate dehydrogenase [NADP] cytoplasmic	−1.07
P10809	*HSPD1*	60 kDa heat shock protein_ mitochondrial	1.02
A0A3B3IUB5	*HM13*	Minor histocompatibility antigen H13	1.02
I3L1P8	*SLC25A11*	Mitochondrial 2-oxoglutarate/malate carrier protein (Fragment)	1.07
A0A3B3IRT8	*SSR1*	Signal sequence receptor subunit alpha	1.09
A0A0G2JIW1	*HSPA1B*	Heat shock 70 kDa protein 1B	1.11
A0A3B3IS40	*KDM5B*	[Histone H3]-trimethyl-L-lysine (4) demethylase	1.12
P07093	*SERPINE2*	Glia-derived nexin	1.12
P11021	*HSPA5*	Endoplasmic reticulum chaperone BiP	1.13
P21796	*VDAC1*	Voltage-dependent anion-selective channel protein 1	1.14
Q70UQ0	*IKBIP*	Inhibitor of nuclear factor kappa-B kinase-interacting protein	1.2
P80303	*NUCB2*	Nucleobindin-2	1.21
A0A590UK15	*NNT*	Proton-translocating NAD(P) (+) transhydrogenase	1.24
P14625	*HSP90B1*	Endoplasmin	1.33
P04264	*KRT1*	Keratin_ type II cyt	1.41
P45880	*VDAC2*	Voltage-dependent anion-selective channel protein 2	1.42
P13645	*KRT10*	Keratin_ type I cyt	1.45
P00403	*MT-CO2*	Cytochrome c oxidase subunit 2	1.46
P13796	*LCP1*	Plastin-2	1.64
A0A6Q8PGJ3	*KIF5A*	Kinesin heavy-chain isoform 5A	1.69
P31689	*DNAJA1*	DnaJ homolog subfamily A member 1	1.79
Q96NL6	*SCLT1*	Sodium channel and clathrin linker 1	1.83
P35908	*KRT2*	Keratin_ type II cyt	1.9
P69905	*HBA1*	Hemoglobin subunit alpha	1.92
O95757	*HSPA4L*	Heat shock 70 kDa protein 4L	2.28
P43251	*BTD*	Biotinidase	4.15
Immune Powder-treated HCoV-229E-infected MRC-5 cells compared to control (HCoV-229E-infected MRC-5 cells)			
E9PFT7	*APC*	Adenomatous polyposis coli protein (Fragment)	−1.97
A0A0U1RRM4	*PTBP1*	Polypyrimidine tract-binding protein 1	−1.82
A0A024R4E5	*HDLBP*	High-density lipoprotein-binding protein (Vigilin)_ isoform CRA_a	−1.55
O15031	*PLXNB2*	Plexin-B2	−1.53
Q13283	*G3BP1*	Ras GTPase-activating protein-binding protein 1	−1.42
O43491	*EPB41L2*	Band 4.1-like protein 2	−1.34
P02452	*COL1A1*	Collagen alpha 1(I) chain	−1.33
Q9UBG0	*MRC2*	C-type mannose receptor 2	−1.32
O76074	*PDE5A*	cGMP-specific 3′_5′-cyclic phosphodiesterase	−1.19
A0A087WTA8	*COL1A2*	Collagen alpha 2(I) chain	−1.18
Q15056	*EIF4H*	Eukaryotic translation initiation factor 4H	−1.11
K7EJ78	*RPS15*	40S ribosomal protein S15	−1.07
P10644	*PRKAR1A*	cAMP-dependent protein kinase type I-alpha regulatory subunit	−1.07
A0A0R4J2E8	*MATR3*	Matrin-3	−1.03
P29966	*MARCKS*	Myristoylated alanine-rich C-kinase substrate	−1.02
P13645	*KRT10*	Keratin_ type I cytoskeletal 10	2.06
P35527	*KRT9*	Keratin_ type I cytoskeletal 9	2.2
P07093	*SERPINE2*	Glia-derived nexin	2.22
A0A0G2JIW1	*HSPA1B*	Heat shock 70 kDa protein 1B	2.32
P35908	*KRT2*	Keratin_ type II cytoskeletal 2 epidermal	2.46
P69905	*HBA1*	Hemoglobin subunit alpha	2.53
A0A6Q8PGJ3	*KIF5A*	Kinesin heavy-chain isoform 5A	2.63
Q96NL6	*SCLT1*	Sodium channel and clathrin linker 1	2.66
P04264	*KRT1*	Keratin_ type II cytoskeletal 1	2.78
P02533	*KRT14*	Keratin_ type I cytoskeletal 14	2.86
P31689	*DNAJA1*	DnaJ homolog subfamily A member 1	3.09
O95757	*HSPA4L*	Heat shock 70 kDa protein 4L	3.77
P43251	*BTD*	Biotinidase	20.99
FMP-treated HCoV-229E-infected MRC-5 cells compared to control (HCoV-229E-infected MRC-5 cells)			
Q5JVD1	*CNTRL*	Centriolin	−4.45
P60709	*ACTB*	Actin_ cytoplasmic 1	−3.85
A0A0U1RRM4	*PTBP1*	Polypyrimidine tract-binding protein 1	−3.77
J3QS41	*HELZ*	Probable helicase with zinc finger domain	−2.91
P30613	*PKLR*	Pyruvate kinase PKLR	−2.48
P10644	*PRKAR1A*	cAMP-dependent protein kinase type I-alpha regulatory subunit	−2.37
A0A0B4J1T8	*EPHA6*	Receptor protein-tyrosine kinase	−2.34
E9PFT7	*APC*	Adenomatous polyposis coli protein (Fragment)	−2.04
P61106	*RAB14*	Ras-related protein Rab-14	−1.97
O43776	*NARS1*	Asparagine--tRNA ligase_ cytoplasmic	−1.96
Q9UBQ7	*GRHPR*	Glyoxylate reductase/hydroxypyruvate reductase	−1.95
Q5JTH9	*RRP12*	RRP12-like protein	−1.92
O75340	*PDCD6*	Programmed cell death protein 6	−1.81
P49221	*TGM4*	Protein-glutamine gamma-glutamyltransferase 4	−1.80
O95302	*FKBP9*	Peptidyl-prolyl cis-trans isomerase FKBP9	−1.78
A0A0R4J2E8	*MATR3*	Matrin-3	−1.74
F8W1A4	*AK2*	Adenylate kinase 2_ mitochondrial	−1.70
Q8IWE2	*FAM114A1*	Protein NOXP20	−1.65
Q15056	*EIF4H*	Eukaryotic translation initiation factor 4H	−1.64
Q13148	*TARDBP*	TAR DNA-binding protein 43	−1.61
A0A024R4E5	*HDLBP*	High-density lipoprotein-binding protein (Vigilin)_ isoform CRA_a	−1.56
P55010	*EIF5*	Eukaryotic translation initiation factor 5	−1.49
A0A0A0MRA5	*HNRNPUL1*	Heterogeneous nuclear ribonucleoprotein U-like protein 1	−1.48
P62736	*ACTA2*	Actin_ aortic smooth muscle	−1.46
Q9UBG0	*MRC2*	C-type mannose receptor 2	−1.42
O43491	*EPB41L2*	Band 4.1-like protein 2	−1.40
O14818	*PSMA7*	Proteasome subunit alpha type 7	−1.35
P52306	*RAP1GDS1*	Rap1 GTPase-GDP dissociation stimulator 1	−1.32
Q09028	*RBBP4*	Histone-binding protein RBBP4	−1.28
A0A6I8PUA5	*AKAP9*	A-kinase anchor protein 9 (Fragment)	−1.26
P02452	*COL1A1*	Collagen alpha 1(I) chain	−1.24
A0A087WTA8	*COL1A2*	Collagen alpha 2(I) chain	−1.23
O94925	*GLS*	Glutaminase kidney isoform_ mitochondrial	−1.20
E7EPK1	*SEPTIN7*	Septin-7	−1.19
Q13283	*G3BP1*	Ras GTPase-activating protein-binding protein 1	−1.19
Q8WX93	*PALLD*	Palladin	−1.19
A0A0A0MSQ0	*PLS3*	Plastin-3	−1.16
P23381	*WARS1*	Tryptophan--tRNA ligase_ cytoplasmic	−1.16
Q5TB53	*TM9SF3*	Transmembrane 9 superfamily member (Fragment)	−1.14
K7EJ78	*RPS15*	40S ribosomal protein S15	−1.11
P42704	*LRPPRC*	Leucine-rich PPR motif-containing protein_ mitochondrial	−1.09
P12109	*COL6A1*	Collagen alpha-1chain	−1.07
Q12841	*FSTL1*	Follistatin-related protein 1	−1.07
P05091	*ALDH2*	Aldehyde dehydrogenase_ mitochondrial	−1.05
P29966	*MARCKS*	Myristoylated alanine-rich C-kinase substrate	−1.05
P06756	*ITGAV*	Integrin alpha V	−1.04
Q13308	*PTK7*	Inactive tyrosine-protein kinase 7	−1.04
Q9Y570	*PPME1*	Protein phosphatase methylesterase 1	−1.03
Q13263	*TRIM28*	Transcription intermediary factor 1-beta	−1.03
A0A087WTP3	*KHSRP*	Far upstream element-binding protein 2	−1.01
P45974	*USP5*	Ubiquitin carboxyl-terminal hydrolase 5	−1.01
Q9H6A9	*PCNX3*	Pecanex-like protein 3	−1.01
P61247	*RPS3A*	40S ribosomal protein S3a	1.02
P78332	*RBM6*	RNA-binding protein 6	1.04
P21980	*TGM2*	Protein-glutamine gamma-glutamyltransferase 2	1.05
Q9Y394	*DHRS7*	Dehydrogenase/reductase SDR family member 7	1.1
O00231	*PSMD11*	26S proteasome non-ATPase regulatory subunit 11	1.11
P13796	*LCP1*	Plastin-2	1.11
A0A0G2JIW1	*HSPA1B*	Heat shock 70 kDa protein 1B	1.2
P02765	*AHSG*	Alpha-2-HS-glycoprotein	1.25
Q9Y4B5	*MTCL1*	Microtubule cross-linking factor 1	1.27
P17050	*NAGA*	Alpha-N-acetylgalactosaminidase	1.31
P02768	*ALB*	Albumin	1.32
A0A6Q8PGJ3	*KIF5A*	Kinesin heavy-chain isoform 5A	1.4
Q96NL6	*SCLT1*	Sodium channel and clathrin linker 1	1.43
P69905	*HBA1*	Hemoglobin subunit alpha	1.61
E7EQB2	*LTF*	Lactotransferrin (Fragment)	1.62
O95757	*HSPA4L*	Heat shock 70 kDa protein 4L	1.84
P80303	*NUCB2*	Nucleobindin-2	1.9
P34932	*HSPA4*	Heat shock 70 kDa protein 4	3.18
P43251	*BTD*	Biotinidase	4.02
Q15811	*ITSN1*	Intersectin-1	4.48

## Data Availability

The relevant data are provided in this paper and the Supplementary File S2. The raw and processed data have also been deposited in the ProteomeXchange Consortium via the PRoteomics IDEntifications (PRIDE) repository with the following dataset identifier: 10.6019/PXD052339, Project accession: PXD052339, and Token: ZMONUGmsYz4r which can be accessed via the following unique link: https://www.ebi.ac.uk/pride/review-dataset/42590c88f30949519fb027b601bc1d9b.

## Supplementary Materials

The following supporting information can be downloaded at: https://www.mdpi.com/article/10.3390/ijms25179353/s1.
