# Supplementary material for: Impact of a Functional Dairy Powder and Its Primary Component on the Growth of Pathogenic and Probiotic Gut Bacteria and Human Coronavirus 229E
Source: Int J Mol Sci. 2024 Aug 29;25(17):9353. doi: 10.3390/ijms25179353 (PMC11394815; doi:10.3390/ijms25179353)
Supplement: Supplementary file 1 [file ijms-25-09353-s001.zip › SUPPLEMENTARY FILE S1.pdf]

Table S1. Constituent of Immune Powder

Ingredient	Supplier	Addition Ratio
Full cream milk powder	Burra Foods Pty Ltd	90%
Natra Fractionated Milk Protein	Saputo Dairy Australia Pty Ltd	3-5%
FOS	BENEO - Orafti	0.1-1.6%
Zinc Sulphate	Vitablend Asia Pacific Pty Ltd	0.1-0.6 %
Maize Maltodextrin	Vitablend Asia Pacific Pty Ltd	0.2-2%
Ferrous Sulphate	Vitablend Asia Pacific Pty Ltd	0.1-0.8%

Table S2. Constituent of Natra Fractionated Milk Protein (FMP)

Ingredient	Ingredient Ratio
Protein (N x 6.38) as is	>95.0%
• Lactoperoxidase % of protein	25.0%-45.0%
• Lactoferrin % of protein	5.0%-20.0%
Immunoglobulin G	5.0% - 10.0%
Immunoglobulin A	0.5% - 1.0%
Immunoglobulin M	0.3% - 0.8%
Moisture	<4%
Ash	<1.5%
Fat	<0.1%
Lactose	<0.1%
pH	5.5-6.5
LPO activity (U/mg)	> 15

Table S3. Preparation of stock solution of Stimulated Gastric Fluid. The volumes are calculated for a final volume of 500 mL.

Constituent	Stock concentration (mol/L)	Volume of stock (mL)	Concentration in Stimulated Gastric Fluid (mmol/L)
KCl	0.5	6.9	6.9
KH_2PO_4	0.5	0.9	0.9
$NaHCO_3$	1	12.5	25

NaCl	2	11.8	47.2
$MgCl_2(H_2O)_6$	0.15	0.4	0.1
$(NH_4)_2CO_3$	0.5	0.5	0.5
HCl	6	1.3	15.6
H2O	-	465.7	-
$CaCl_2(H_2O)_2$	0.3	Added to the final mixture of Stimulated Gastric Fluid to archive concentration of 0.15 mmol/L	0.15

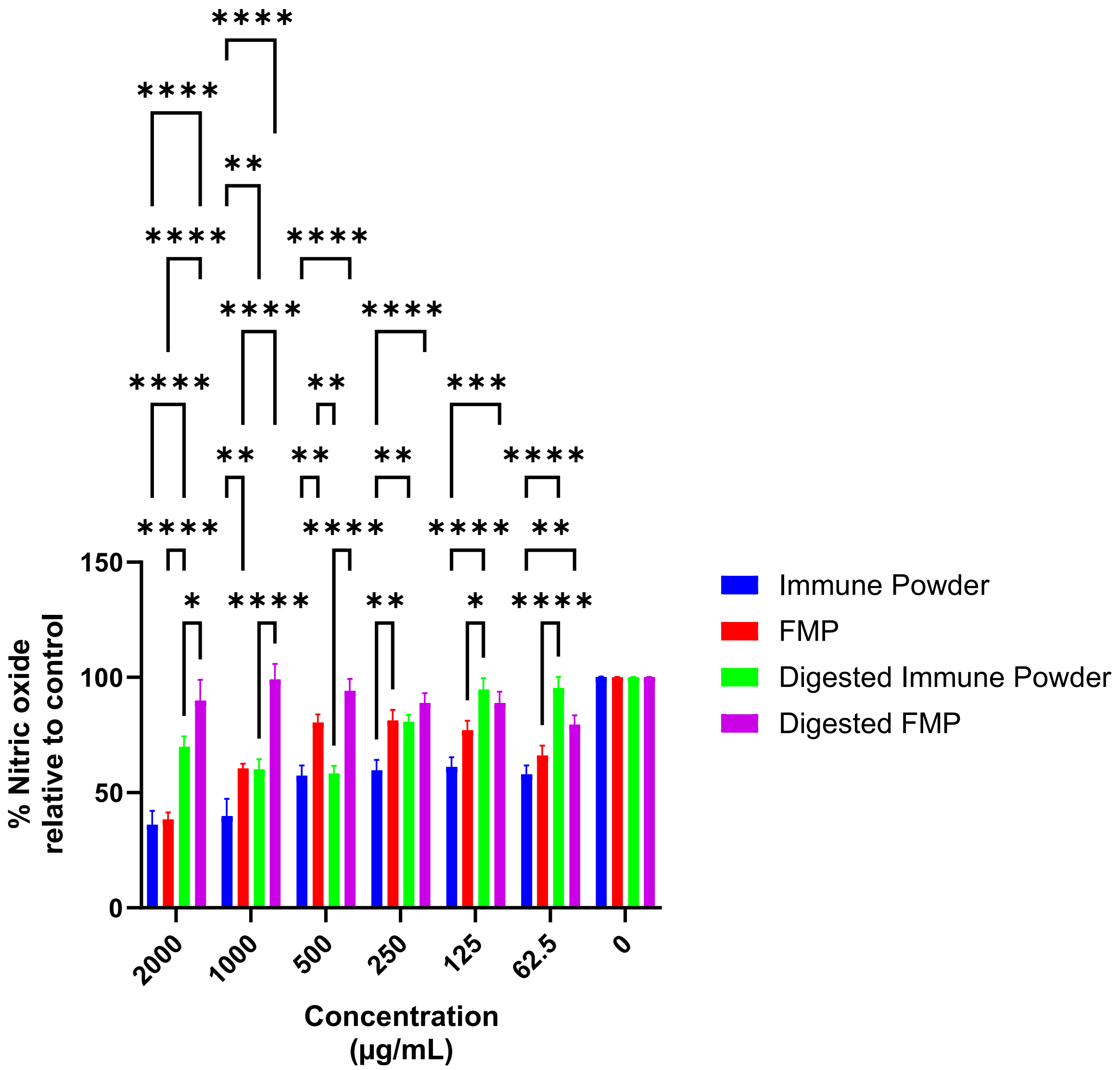

Figure S1. NO production relative to the control with Two-way ANOVA statistical analysis of significant pairs $p < 0.05$.
